# 减轻肺叶切除术后急慢性疼痛，“单孔”比“三孔”更有优势么？

**DOI:** 10.3779/j.issn.1009-3419.2018.04.10

**Published:** 2018-04-20

**Authors:** 鹤成 李, 亚杰 张

**Affiliations:** 上海交通大学医学院附属瑞金医院胸外科 Department of Thoracic Surgery, Ruijin Hospital Affiliated to Shanghai Jiao Tong University

胸外科手术后急性与慢性疼痛的发生与严重程度是患者术后康复的重要影响因素，直接影响患者咳嗽、咳痰及早期活动，严重者可造成肺不张、肺部感染、低氧和二氧化碳蓄积等并发症^[1]^。自20世纪90年代开始，电视胸腔镜技术逐渐应用于胸外科疾病的诊治^[2]^，目前胸腔镜肺叶切除+纵隔淋巴结清扫或系统淋巴结采样术已成为早期肺癌的标准手术式^[3]^。与传统开放手术相比，胸腔镜手术具有创伤小、术后疼痛轻及并发症发生率低等优势^[4]^，但仍有超过50%的患者有术后疼痛或胸壁感觉异常等不适^[5]^。近年来由于手术方式的改进及手术器械的发展，胸腔镜手术的入路逐渐由四孔法到三孔法，再到双孔法甚至发展到单孔法。2004年Rocco等^[6]^首次报道了单孔胸腔镜肺楔形切除术，2011年Gonzalez等^[7]^首次报道了单孔胸腔镜下肺叶切除+系统性淋巴结清扫术。目前单孔胸腔镜技术得到迅速发展，几乎涵盖了传统胸腔镜肺癌手术的所有术式^[8]^。研究表明单孔胸腔镜肺叶切除术手术安全、可行，并可以达到肿瘤根治的效果^[9]^。与传统三孔胸腔镜肺叶切除术相比，单孔胸腔镜手术去除了副操作孔和观察孔，在患侧胸壁第4或5肋间腋中线与腋前线之间作单个切孔，手术操作难度更大，对术者技术要领及器械设备要求更高，但其是否能进一步减少手术创伤、减轻患者术后急慢性疼痛目前仍存有争议^[10]^。有研究报道单孔胸腔镜肺叶切除术后患者急性疼痛评分低于三孔传统胸腔镜组^[11]^。然而一项关于单孔胸腔镜与三孔胸腔镜手术术后疼痛对比的meta分析结果提示单孔手术在减轻术后疼痛方面并无优势，但其研究对象混杂包括有气胸、肺叶切除等多种手术方式^[10]^。目前关于单孔胸腔镜对术后疼痛影响的研究均为回顾性研究，入组例数较少，且多集中于急性疼痛的研究。

本项目旨在对比研究单孔与三孔胸腔镜肺叶手术对肺癌患者术后急慢性疼痛的影响。研究对象为131例单孔与101例三孔胸腔镜肺叶切除手术患者，以数字评定量表（numeric rating scale, NRS）评估急性和慢性疼痛的发生情况。作者通过对比发现与三孔胸腔镜手术相比，单孔胸腔镜肺叶切除术在患者术后急性和慢性疼痛发生方面均存在优势，两组之间其余围手术期结果无明显差异。通过进一步分析术后慢性疼痛发生的危险因素，发现手术方式、手术时间及术后14天急性疼痛程度是慢性疼痛的危险因素。本研究对广大胸外科同道们具有借鉴作用，不仅表明与传统三孔胸腔镜肺叶切除手术相比，单孔胸腔镜手术在减轻肺癌患者术后急慢疼痛方面具有优势，并进一步提示我们优化单孔胸腔镜手术的技术与流程，在保证安全、根治的前提下有效缩短手术时间，可减少患者慢性疼痛的发生率。

本次临床研究为回顾性对比研究，各组入组例数较多，研究内容详细，包括了单孔和三孔胸腔镜肺叶切除术后的急慢性疼痛指标及慢性疼痛的危险因素，对临床工作具有指导意义。同时本研究也受制于未进行组间匹配及单中心、回顾性研究等局限性。期待关于单孔胸腔镜与传统三孔胸腔镜手术方式对患者术后疼痛影响的随机对照临床研究的开展。
